# *In vitro* antiviral activity against rotavirus and astrovirus infection exerted by substances obtained from *Achyrocline bogotensis* (Kunth) DC. (*Compositae*)

**DOI:** 10.1186/s12906-015-0949-0

**Published:** 2015-12-03

**Authors:** M. A. Téllez, A. N. Téllez, F. Vélez, J. C. Ulloa

**Affiliations:** Laboratorio de Virología, Departamento de Microbiología, Facultad de Ciencias, Grupo de Enfermedades Infecciosas, Pontificia Universidad Javeriana, Cra. 7 # 43-82, Bogotá, Colombia; Laboratorio de Fitoquímica, Departamento de Química, Facultad de Ciencias, Grupo de Investigación en Fitoquímica, Pontificia Universidad Javeriana, Bogotá, Colombia; Departamento de Matemáticas, Grupo de Investigación Signos, Universidad El Bosque, Bogotá, Colombia

**Keywords:** Antiviral, Rotavirus, Astrovirus, *Achyrocline bogotensis*

## Abstract

**Background:**

*Achyrocline bogotensis* has been traditionally used to treat infections of skin, respiratory, tract urinary and other infections, but not to treat viral gastrointestinal disease. In this study, this Colombian native medicinal plant was investigated by its *in vitro* anti-rotavirus and anti-astrovirus activity.

**Methods:**

Several extracts and fractions phytochemically obtained from *A. bogotensis* were evaluated initially for their cell toxicity on MA104 and Caco2 cells and then for their anti-rotavirus (RRV) and anti-astrovirus (Yuc8) activity following three strategies: pre-treatment of cells (blocking effect), direct viral activity (virucidal effect) and post-treatment of infected cells (reduction of viral yield post-infection). In addition qualitative chemical studies were developed for the active compounds.

**Results:**

Non-toxic concentrations of a fraction obtained exhibited antiviral activity against both viruses characterized by a virucidal effect and by the reduction of the infectious particles produced post-infection. Steroids, sterols, terpenes, phenols, flavonoids and sesquiterpenlactones were identified qualitatively in the active fraction.

**Conclusions:**

*A. bogotensis* contains substances with *in vitro* antiviral activity against rotavirus and astrovirus. This study confirms their anti-microbial properties and describes by the first time its antiviral activity *in vitro*.

## Background

*Achyrocline bogotensis* (Kunth) DC. (*Compositae*) is an endemic plant of Colombia known with the vernacular names *vira vira*, *cenizo* and *suso*. This species is traditionally used in decoction to treat skin diseases, chronic cough and as expectorant, and in infusion to treat prostatitis, kidney pain, inflammatory processes and to clean the urinary tract [1]. Additionally, anti-cancer activity of flavonoids obtained from *A. bogotensis* was reported *in vitro* on several cell lines [2]. Its *in vitro* activity against bacteria and fungi has been described [1], but there are no reports on antidiarrheal properties or antiviral activity of substances extracted from this medicinal plant.

Rotavirus belongs to the family *Reoviridae,* which includes naked icosahedral viruses with three concentric shells of approximately 70 nm in diameter and double-stranded ribonucleic acid (dsRNA) divided into 11 gene segments that encode five non-structural and six structural proteins. Rotavirus infects a wide variety of mammals included humans. It is the most common cause of severe gastroenteritis in infants and young children worldwide, who acquire the virions by direct fecal-oral route or by fecally contaminated food and water, and respiratory droplets [3, 4].

Astrovirus (AstVs) belongs to the family *Astroviridae* and infects mammals (*Mamastrovirus*) and birds (*Avastrovirus*) [5–8]. Astroviral genome (ssRNA positive) is divided into three open reading frames (ORF1a, ORF1b and ORF2) which encode two non-structural proteins and one structural polyprotein, respectively [8]. This virus has been associated mainly with gastroenteritis in mammals and hepatitis and fatal nephritis in birds [9]; nevertheless, since 2010 it has been associated with neurological disorders in humans, minks and cows [10–12].

Diarrhea remains the second leading cause of death among children under five globally [13]. Viruses are the major etiological agents of acute gastroenteritis (AGE) in this population. The most frequent enteropathogenic viruses associated with AGE are rotavirus, norovirus, enteric adenovirus, astrovirus and saporovirus [14], being all transmitted by the fecal-oral route. Rotavirus alone causes annually two million hospitalizations and 500 deaths in developing countries [15]. Several reports have described the prevalence of the rotavirus as the single cause of diarrhea in children; however, it is well known that this virus can also be found co-infecting along with other viruses as astrovirus [16–18].

At present, the viral diarrhea treatment focuses on fluid replacement with oral rehydration salts (ORS) to prevent dehydration and zinc treatment to decrease its severity and duration. So far, no specific antiviral drug is available to treat the rotavirus infection [19]. In the case of astrovirus, lack of understanding its pathogenesis has delayed the search of effective antiviral substances.

The difficulty in developing new antiviral agents is a significant challenge. Nowadays, there are several problems that must be overcome such as the limited availability of new synthetic molecules, the toxicity exerted on the host and the resistance exhibited by new mutant viruses. One alternative is the use of new compounds that can be isolated from medicinal plants, which have traditionally been used for centuries.

In order to shed light on a potential use of *A. bogotensis* in controlling infections produced by enteropathogenic viruses as rotavirus and astrovirus, we evaluated the *in vitro* antiviral activity of substances obtained from this medicinal plant using three strategies. Low non-toxic concentrations of a fraction phytochemically obtained showed a significant virucidal effect and a reduction of the number of infectious particles produced post-infection. These results suggest another potential use of *Achyrocline bogotensis*.

## Methods

### Plant collection

Five kg of aerial material (leaves and stems) from adult plants were collected for the purposes of this study and for verifying the taxonomic identification by botanist Henry Yesid Bernal of the herbarium of the Pontificia Universidad Javeriana (HPUJ) - Bogotá, Colombia. Voucher specimens were deposited at HPUJ. Plant material collected for the study was taken to the Phytochemistry Laboratory of the same university.

The collection and processing of wild material was covered by the collection permission number 1439 issued by the National Environmental Licensing Authority – ANLA) and the Contract No. 084 on genetic resource access issued by the Ministry of Environment and Sustainable Development of Colombia.

### Liquid-liquid fractionation and screening of antiviral compounds

The leaves and stems of *A. bogotensis* were initially dried at room temperature (RT) and then washed using chloroform and hexane prior the obtainment of E1 and E2 extracts, respectively. Then, the washed leaves and stems were cold soaked with ethanol to obtain the E3 extract. The E3 extract was fractioned by continuous liquid/liquid fractionation with hexane, dichloromethane and ethyl acetate for obtaining the F1, F2 and F3 fractions, respectively. All the extracts and fractions were placed in a rotary evaporator at 40 °C to concentrate the substances.

For further testing of biological activity, the extracts and the fractions were dissolved in dimethyl sulfoxide (DMSO, Merck/Cat. # 317275) to a final concentration of 100 mg/ml (stock solution). Serial dilutions 1:2 were prepared ranging from 1.000 μg/ml down to 0 μg/ml for each extract and fraction in Advanced DMEM (Dulbecco’s Modified Eagle Medium Gibco®/Cat # 12491-023) supplemented with 2 mM L-glutamine and antibiotic/antimycotic, without fetal bovine serum (FBS).

### Cell lines

MA104 and Caco2 cells were cultured in Advanced DMEM supplemented with 4 and 5 % Fetal Bovine Serum (FBS) respectively, 2 mM L-glutamine and antimicotic/antibiotic in a 5 % CO_2_ atmosphere at 37 °C.

### Cell toxicity assays

Ten thousand MA104 and fifteen thousand Caco2 cells were seeded in separate wells in 96 multiwell plates and were cultured for 24 and 72 h respectively, as mentioned above. At this time, the medium was removed, the cells washed once with Phosphate Buffer Saline (PBS), and 100 μL of several dilutions 1:2 (from 1.000 μg/mL to 0 μg/mL) of each extract and fraction (solubilized previously in DMSO at a final concentration of 100 mg/mL) diluted in Advanced DMEM without FBS. Each resulting mixture was applied to MA104 and Caco2 cells by triplicate for 96 h. After this time, cell viability was evaluated using firstly trypan blue exclusion assay by removing the culture medium and adding 50 μL per well of trypan blue (0.4 % in sterile PBS pH = 7.2). Then, three random fields per well were observed and each one recorded photographically. Secondly, the MTT (3-(4,5-dimetiltiazol-2-il)-2,5-difeniltetrazolio – Sigma-Aldrich®/Cat# M5655) method was followed according to the manufacturer’s instructions. Optic densities were measured at 492 nm in a Multiskan Reader MCC/340 Labsystems. Additionally, in order to know the toxic effect of the solvent used, several concentrations of DMSO (0, 0.5, 1, 1.5, 2, 2.5, 3.0, 3.5, 4.0, 4.5 and 5 %) were also evaluated in the same way. All assays were done three times independently by triplicate.

### Viruses and antibodies

Rhesus rotavirus (RRV strain) and human astrovirus serotype 8 (HAstV-8 Yuc8 strain) were multiplied in MA104 or Caco2 cells, previously cultured as mentioned above. For this, 10 and 200 μg/ml porcine pancreatic trypsin (Sigma-Aldrich®/Cat# T-4799) were added to RRV and Yuc8 respectively, during 1 h at 37 °C in order to activate viral particles. After this time, soybean inhibitor (Life Technologies®/Cat# R-007-100) was added for 5 min at RT. Then, the activated viruses were added (adsorption period) to each cell line respectively, during 1 h in a 5 % CO_2_ atmosphere at 37 °C. After one wash with PBS, fresh culture medium without FBS was added to complete 10 (RRV) and 12 (Yuc8) hour post-infection (hpi) respectively, until the immunodetection test (See below).

Anti-TLP (Triple – Layered Particles) polyclonal antibodies donated by Dr. Carlos Arturo Guerrero from Universidad Nacional de Colombia or anti-Yuc8 polyclonal antibodies donated by Dr. Ernesto Méndez^†^ from Instituto de Biotecnología – Universidad Nacional Autónoma de México were used to detect cytoplasmic viral antigens by immunocytochemistry or flow cytometry. The conjugates and substrates used were peroxidase – goat anti-rabbit IgG (Invitrogen™/Cat# 65-6120), AEC (3-Amino-9-ethylcarbazole – Sigma-Aldrich®/Cat# A5754) with hydrogen peroxide 0.02 %, and Alexa Fluor® 488 Goat (Life Technologies/Cat# A11034), respectively.

### Anti-rotavirus and anti-astrovirus activity assays

In order to determine if the extracts and fractions obtained can exert an anti-rotavirus and anti-astrovirus activity, three strategies were evaluated based on a blocking effect, a virucidal effect or a reduction of infectious particles produced post-infection (viral yield). In addition, to develop these strategies a bio-guided assay was carried out according to the results of each strategy.

#### Blocking effect assay

MA104 or Caco2 cells previously cultured in 96 multi well plates as mentioned before were covered for 24 h at 37 °C in a 5 % CO_2_ atmosphere with several non-toxic concentrations of each of the extracts or fractions diluted in culture media without FBS. Then substances were removed and the cells washed two times with PBS and challenged with rotavirus – RRV strain – or astrovirus – Yuc8 strain – at a multiplicity of infection (MOI) of 1, previously activated with 10 μg/mL or 200 μg/mL of trypsin respectively, at 37 °C for 1 h. After this viral adsorption period, the inoculum was removed, the cells washed once with PBS, and fresh culture media without SFB was added to complete 10 (RRV) or 12 (Yuc8) hpi before cells were processed by immunocytochemistry (See below). DMSO and infected or uninfected cells (MOCK) were included as controls of the activity and viral infectivity. All experiments were carried out three times independently by triplicated.

#### Virucidal effect assay

The same concentrations evaluated in the above experiments from each extract and fraction were co-incubated with the previously trypsin activated rotavirus or astrovirus at a final MOI of 5 (RRV) or 1 (Yuc8) for 1, 2 and 4 h at 37 °C in a 5 % CO_2_ atmosphere. Then, each of the mixtures was placed on MA104 or Caco2 cells for 1 h at 37 °C in a 5 % CO_2_ atmosphere. The inoculum was removed, the cells washed once with PBS, and fresh culture medium without SFB was added to complete 10 (RRV) or 12 (Yuc8) hpi, before being processed by immunocytochemistry. Viruses without treatment and viruses with DMSO were included as controls of the activity and viral infectivity. All experiments were carried out three times by triplicated.

#### Viral yield reduction assay

MA104 and Caco2 cells were cultured in 96 multi-well plates as mentioned before. Then, each cell line was infected with rotavirus and astrovirus at MOI of 1, previous viral activation with trypsin for 1 h. After the adsorption period, the inoculum was removed, the cells washed once with PBS and the same concentrations of each extract and fractions diluted in culture media without SFB were added to complete 24 h at 37 °C in a 5 % CO_2_ atmosphere. At this time the viruses were harvested by freezing and thawing twice, centrifuged at 16,000 × g for 1 min and the supernatant recovered. The focus forming units per milliliter (FFU/mL) of rotavirus and astrovirus present in the viral suspension were then quantified by immunocytochemistry. Infected or mock infected cells without treatment were included as controls of the assay. Quantifications were performed three times independently by triplicated.

### Determination of the antiviral activity

#### Immunocytochemistry

MA104 and Caco2 cells were cultured in 96 multi-well plates as mentioned above. After 10 or 12 hpi with RRV or Yuc8 respectively, the culture medium was removed and 80 % acetone in PBS was added for 15 min at RT. Two washes with PBS were performed and 50 μl of anti-TLPs 1:3000 or anti-Yuc8 1:5000 respectively, were added per well and incubated for 1.5 h at 37 °C in moist chamber. After three washes with PBS, 50 μl of the conjugate was added for 1 h at 37 °C in moist chamber. Finally, the conjugate was removed, cells were washed three times, and AEC (3-Amino-9-ethylcarbazole) with 0.02 % hydrogen peroxide was added to verify cytoplasmic brown staining. The reaction was stopped with two water washes and the focus forming units per milliliter (FFU/mL) were quantified and photographic records of three random fields per well were obtained with an Olympus CKX41 inverted microscope.

#### Flow cytometry assay

Virucidal activity was quantified using flow cytometry in a Becton Dickinson FACSAria II. After the treatments of cells or viruses at a MOI of 5 (RRV) or 2 (Yuc8), cells were detached and fixed with 2 % paraformaldehyde for 15 min at RT. After two washes with PBS, the cells were permeabilized with 0.03 % Triton X-100 for 15 min at RT and anti-TLPs or anti-Yuc8 polyclonal antibodies prepared in PBS containing 0.03 % Triton X-100 and 5 % FBS were added for 1.5 h at RT. After this time, two washes were done and Alexa Fluor 488® was added in the dark for 1.5 h at RT. Finally, three washes were done and the percentages of infected cells were obtained. These assays were performed three times independently by duplicate.

#### Preliminary phytochemical characterization of antiviral substances

In order to make an approximation to the composition of the extracts or fractions with antiviral activity in this study, we carried out the following qualitative chemical tests: Liebermann-Burchard, ammonium molybdate, ferric chloride, Dragendorff reagent, Bornträger, Shinoda, Salkowski, Baljet, Molisch and foam index.

#### Statistical analysis

Analysis of variance (ANOVA) for repeated measures and univariate analysis were applied using the SPSS software version 21 (SPSS Inc., Chicago, IL). The significance for these analyses was *P* < 0.05.

## Results

### Cytotoxicity exerted by the extracts and fractions obtained on MA104 and Caco2 cells

**Fig. 1:**
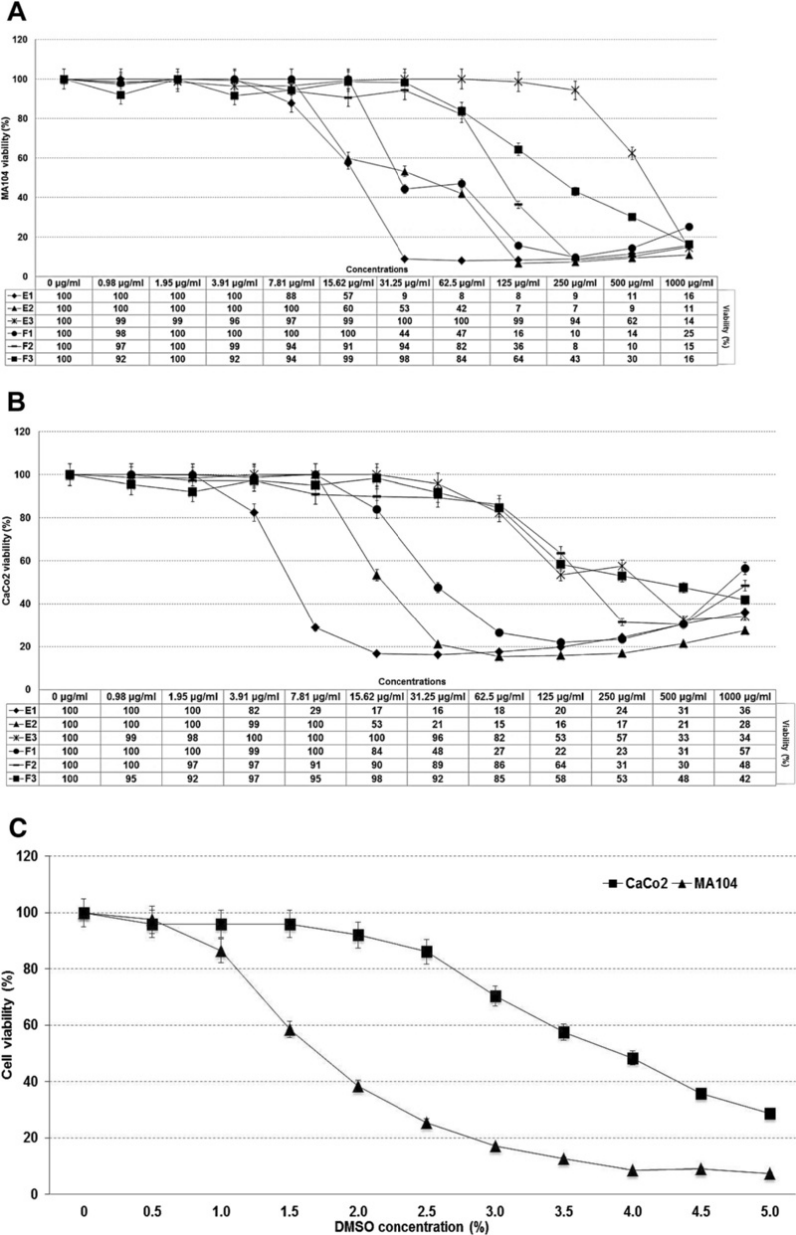
Viability of MA104 (**a**) and CaCo2 (**b**) cells in the presence of extracts, fractions and DMSO (**c**) measured by the MTT (DO_492nm_) method. Table under the X axis (Figures **a** and **b**) corresponds to the percentage (%) of cell viability obtained

Several concentrations of three extracts and three fractions phytochemically obtained were tested by their toxic effect on MA104 and Caco2 cells by trypan blue exclusion assay and MTT assay for 96 h. The results obtained with both tests were consistent, because those concentrations that allowed obtaining high cell viability percentages by the MTT method, also showed kept the cell membrane structure using trypan blue stain. Figure 1 shows the viability percentages obtained with the MTT method on MA104 (Fig. 1a) and Caco2 (Fig. 1b) cells. In general, the most cytotoxic substances were found in the extracts E1, E2 and the fraction F1. The maximum non-toxic concentrations for each extract and fraction were determined and defined as those concentrations which allowed viabilities greater than 95 %. In addition, the toxicity exerted by the DMSO (Fig. 1c) showed that concentrations lower than 0.5 % allowed a viability greater than 96 % in both cell lines during 96 h, while concentrations greater than 0.5 % exerted differential toxic effects; concentrations higher than 1 % were considerably more toxic on MA104 cells than on Caco2 cells.

### The fraction F1 from *A. bogotensis* at non-cytotoxic concentrations exerts an anti-rotavirus and anti-astrovirus activity

**Fig. 2:**
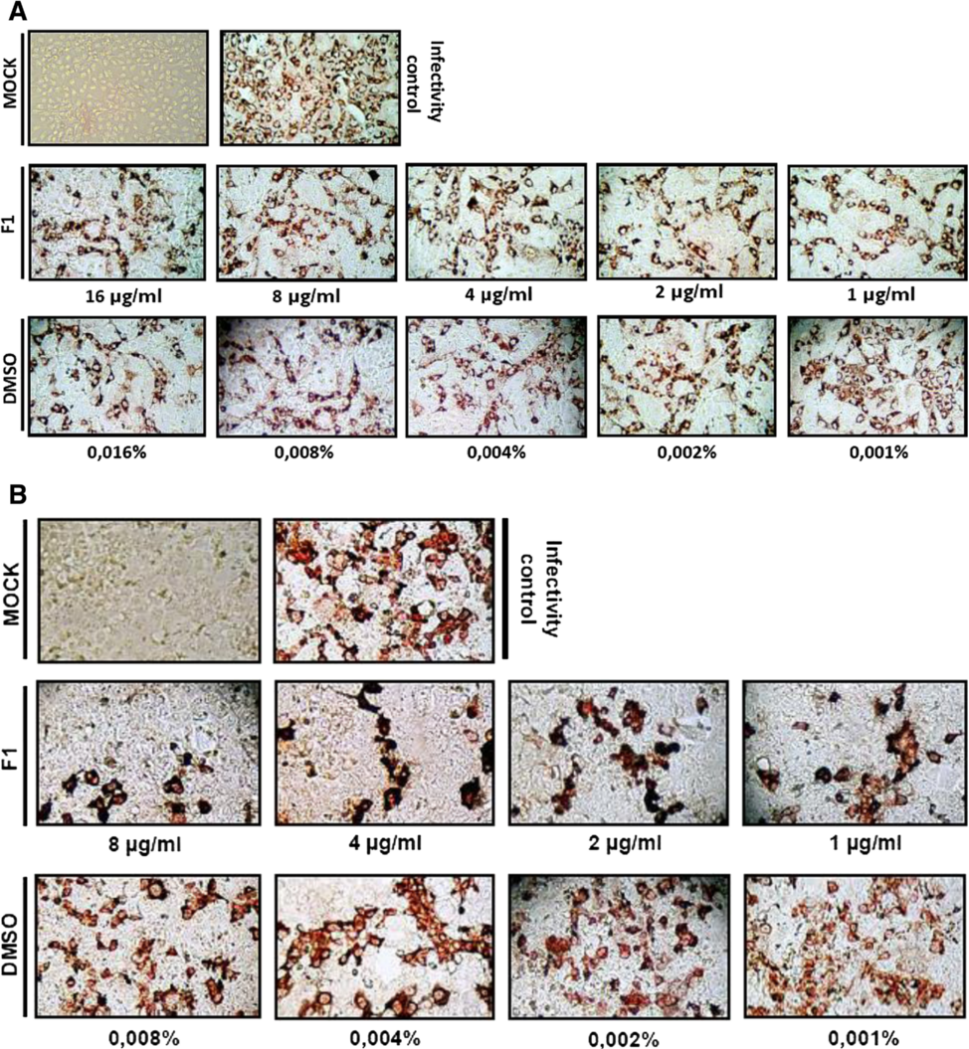
Evaluation of the virucidal effect exerted by F1 fraction from *Achyrocline bogotensis* (Kunth) DC. (Compositae) on (**a**) rotavirus (RRV strain/MOI = 5) and (**b**) astrovirus (Yuc8 strain/MOI = 2). Viruses were first co-incubated with several nontoxic concentrations of F1 during 1 h at 37 °C, and then mixtures were placed on Ma104 and Caco2 cells, respectively. At 10 or 12 hpi intracellular virions were immunodetected in order to find a decrease of the infectivity. Viruses co-incubated with the corresponding concentrations of the solvent of F1 (DMSO), were used as controls. The pictures were taken with a 10X objective in an Olympus CKX41 microscope

The antiviral activity exerted by different non-toxic concentrations of the extracts and fractions obtained from *A. bogotensis* in the study against rotavirus and astrovirus was evaluated considering (1) a possible blocking of viral receptors, (2) a virucidal activity, or (3) a decrease in the production of infectious viral particles after the replicative cycle. Each experiment included an activity control using the solvent of the substances evaluated (DMSO) present in each extract or fraction. For the first strategy, several dilutions 1:2 of all extracts and fractions from the maximum non-toxic concentrations previously determined were placed on the cells for 24 h prior rotavirus or astrovirus infection. The results showed no blocking effect of the viral infection (Data not shown) by immunocytochemistry test. Then, the same dilutions of the substances were co-incubated with infectious particles of rotavirus (MOI of 5) – RRV strain – or human astrovirus serotype 8 (MOI of 2) – Yuc8 strain, previously activated with trypsin. Only the fraction F1 showed a virucidal effect on RRV and Yuc8. Figure 2 shows the effect observed initially by immunocytochemistry with RRV (Fig. 2a) and Yuc8 (Fig. 2b) co-incubated during one hour with F1 fraction on MA104 and CaCo2 cells, respectively. Simultaneously and unexpectedly, it was found that the low DMSO concentrations (≤0.016 %) present in the different dilutions of the tested fraction exhibited a similar effect on the two viruses, with a greater activity against RRV.

**Fig. 3:**
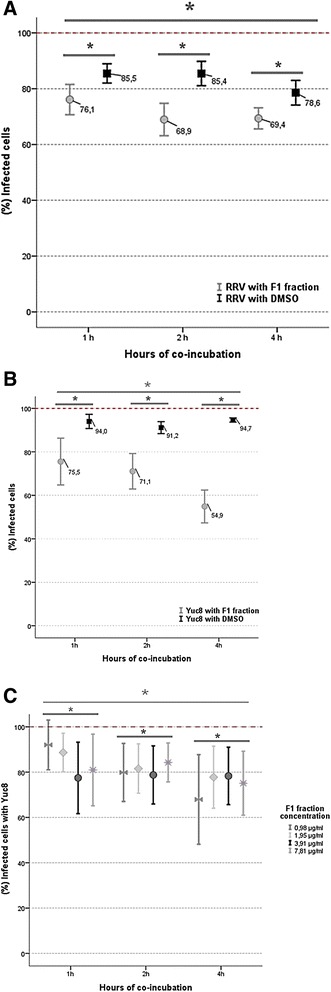
Statistical analysis of the virucidal effect exerted by the F1 fraction on rotavirus (RRV strain/MOI = 5) (**a**) and astrovirus (Yuc8 strain/MOI = 2) (**b**, **c**) by flow cytometry. Viral particles were co-incubated with F1 during 1, 2 and 4 h at 37 °C. The data represent the percentage (%) of infected cells, the bars represent the standard error of the mean (SEM) of three independent experiments by duplicated using flow cytometry. *p* ≤ 0.05 (ANOVA with repeated measures - RM)

In order to confirm and quantify the virucidal effect exerted by F1 and DMSO, flow cytometry was developed from the co-incubation during 1, 2 and 4 h of the fraction with RRV (MOI of 5) and Yuc8 (MOI of 2). The results confirmed the virucidal effect of F1 with all concentrations (0.98 to 15.6 μg/ml) and times tested; additionally, 3.91 μg/ml was the concentration that exhibited the greatest virucidal activity; thus, this concentration of F1 co-incubated for 1, 2 and 4 h with RRV reduced the viral infectivity by 33.6, 30.5 and 41.8 %, respectively, while with Yuc8 reduced the infectivity by 33, 24.4 and 46 %, respectively. The statistical analysis of this effect on RRV using analysis of variance (ANOVA) for repeated measures with an error probability of 0.05 (*p* <0.05), revealed the existence of statistically significant differences between the times tested (*p* = 0.039), confirming that prolonged exposure of RRV particles to the fraction F1, exerts a greater virucidal effect (Fig. 3a); additionally, it was confirmed the significance of the virucidal effect of F1 in comparison with DMSO on RRV infectious particles (*p* = 0.000), which is time-dependent.

Moreover, the statistical analysis of the virucidal effect exerted by the F1 fraction co-incubated during 1, 2 and 4 h with Yuc8 (Fig. 3b) confirmed significant differences between the times studied (*p* = 0.011). Furthermore, the analysis also showed significant differences between the effect produced by the F1 fraction and the DMSO (*p* = 0.000), being greater with F1, and it was also revealed that the interactions of the variables exposure time and concentration, and exposure time and substance (F1 or DMSO) have significant differences (*p* = 0.03 and *p* = 0.02 respectively), confirming that the effect of F1 on Yuc8 is dependent of the time and dose (Fig. 3c), and in turn that the DMSO effect is significantly less.

**Fig. 4:**
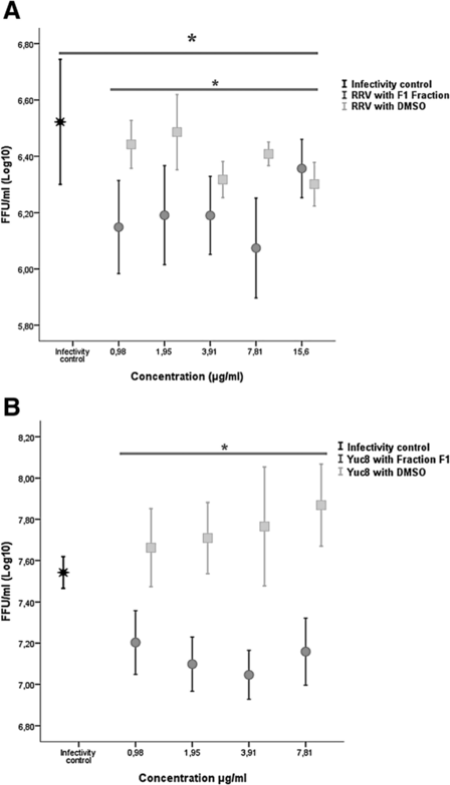
Statistical analysis of the anti-rotavirus (**a**) and anti-astrovirus (**b**) activity exerted by the F1 fraction on the reduction of infectious viral particles produced post-infection in vitro. The graphic shows the focus forming units (FFU/ml) plus standard error of the mean (SEM) from three independent experiments. * *p* ≤ 0.05 (univariate ANOVA)

Finally, the third strategy was to evaluate whether the substances obtained exert post-infection antiviral activity against rotavirus and astrovirus by reducing the infectious viral yield. For this, infected cells (MOI of 1) were treated with different concentrations of F1 fraction and the FFU/ml of RRV and Yuc8 was quantified by immunocytochemistry. The results showed again that the F1 fraction has antiviral activity against rotavirus and astrovirus when it is added immediately after virus adsorption. Figure 4 shows the statistical analysis of the effect using univariate ANOVA with an error probability of 0.05 (*p* <0.05) which showed significant differences between the substances evaluated (Fraction F1 and DMSO) on RRV and Yuc8 (*p* = 0.001 and *p* = 0.000, respectively) confirming the significant decrease of the viral infectious progeny by F1 on RRV infected cells (Fig. 4a). By the contrary, no statistically significant effect on the infectious particle yield of Yuc8 was observed with DMSO (Fig. 4b), but a significant reduction with F1 (*p* = 0000) was detected. The F1 concentrations assessed showed no statistically significant differences.

### Phytochemical partial characterization of F1 fraction

The qualitative chemical tests performed showed that the fraction F1 contains steroids, sterols, terpenes, phenols, flavonoids and sesquiterpenlactones.

## Discussion

The vademecum of medicinal plants of Colombia [1] includes 127 species among which 40 are native and two are endemic. Particularly, *Achyrocline bogotensis* is one of these endemic species which has been traditionally used to treat prostatitis, kidney pains, inflammatory processes and to clean the urinary tract [1, 20]. In Colombia a phytopharmaceutical form is currently approved as co-adjuvant in the treatment of mild inflammation of the urinary tract. To date there are no reports of studies designed to assess the antiviral activity of this medicinal plant; however, other species of the same genus such as *A. satureioides* and *A. flaccida* have been reported by their antiviral activity against Human Herpes Virus [21–23], Human Immunodeficiency Virus [24] and Western Equine Encephalitis Virus [25].

In this study for the first time the anti-rotavirus and anti-astrovirus activities exerted by extracts and fractions obtained from *A. bogotensis* were investigated using three *in vitro* strategies to make an approximation of their potential use in vivo. Although an antiviral effect mediated by the blocking of cellular receptor molecules involved in the entry of RRV and Yuc8 was not found, a virucidal effect and a decrease of the viral yield were confirmed using the F1 fraction. Nevertheless, other studies have shown that natural compounds isolated from licorice root (*Glycyrrhiza glabra*) have the ability to inhibit rotavirus infection *in vitro* used as a pre-treatment [26, 27]. However, previous phytochemical studies carried out with *A. bogotensis* demonstrated the presence of flavonoids [28] which have been extensively studied by their antiviral properties, including the inhibitory effect on rotavirus infection [29, 30]. Qualitative tests carried out in this work aimed to a partial characterization of the active F1 fraction confirmed the presence of flavonoid compounds.

The main target organ of rotaviruses and astroviruses is the small intestine, where their capsid proteins undergo a trypsin-dependent cleavage to enhance the entry to the mature enterocytes [9, 31]. The virucidal activity of the F1 fraction demonstrated in this study has a significant direct effect against the rotavirus (RRV) and astrovirus (Yuc8) particles after a process of trypsinization prior to the infection *in vitro*, suggesting that some or all of the substances contained in this fraction have the ability to bind unspecifically or to interact directly with the structural viral proteins responsible for the adhesion to the host cell, thus avoiding its infection. A similar behavior was reported by Takahashi et al. in 2001, who identified a polysaccharide obtained from another species of the family Asteraceae (*Stevia rebaudiana*) with anti-rotavirus activity. This effect is provided by the ability of the polysaccharide to adhere to the VP7 glycoprotein, decreasing the adhesion of the virus to the cells; further evidence suggests that the same effect may be exerted on VP4 [32]. Another study reported likewise the virucidal activity of extracts obtained from *Alpinia katsumadai* (Zingiberaceae) when they were co-incubated for one hour with porcine rotavirus (G5 P[7]) and bovine rotavirus (G8 P[7]) [33].

Similarly, the results of this study show that the F1 fraction exerted a significant virucidal activity against the astrovirus (Yuc8 strain) depending on the exposure time and fraction concentration. This is the first report of substances of plant origin with virucidal activity against astrovirus. Like rotavirus, astroviruses are naked viruses that require prior trypsin-dependent cleavage to enhance their infectivity [34]. Based on our results it is possible to speculate that the interaction occurred among substances contained in F1 is virus-nonspecific, since it has been described that, for example, flavonoids have the ability to bind to viral surface proteins and thus prevent the infection [35].

Moreover, DMSO which was used as diluent of the extracts and fractions unexpectedly showed a virucidal effect against the particles of rotavirus and astrovirus. This is an interesting finding since the concentrations of DMSO that we used were so reduced (lower than 0.016 %); however the antiviral effect exhibited by the DMSO was significant lower than the activity showed by the F1 fraction solubilized with this solvent (Fig. 3a and b). Although the virucidal effect of DMSO was reported very early [36], this study also reports for the first time this effect against rotavirus and astrovirus. DMSO is widely used in the evaluation of biological activity of plant substances [37–39]. Its low toxicity in cell culture renders it an excellent solvent for such studies [40]. Additional studies to elucidate the effect produced by the F1 fraction and DMSO on rotavirus and astrovirus structures are required.

The last strategy used to evaluate the antiviral activity of F1 was its addition post-infection, immediately after the viral adsorption period, measuring the infectious particles produced after a replicative cycle. Again, the significant anti-rotavirus and anti-astrovirus activities were observed with the F1 fraction; however, particularly on Yuc8, the DMSO treatment showed a stimulation of the viral yield in comparison with infected cells without further treatment. A similar effect was reported by Scholtissek and Muller, where “DMSO by itself had a certain stimulating effect on virus replication” using New Castle Virus and Semliki Forest Virus [40]. To date, only one report has described the post-infection anti-astrovirus activity of different extracts from 17 medicinal plants in Nigeria against animal and human viruses *in vitro*. The results revealed the inhibitory action of the cytopathic effect of astrovirus when the extracts were placed on cells after viral adsorption [41]. However, this effect was detected at extract concentrations of 1 and 2 mg/ml, which are much higher than those concentrations evaluated in our work.

## Conclusions

In conclusion, this study reports for the first time the antiviral activity exerted by an active fraction obtained from *A. bogotensis* on rotavirus and astrovirus infection *in vitro*. The results showed suggest another possible natural alternative to treat rotavirus and astrovirus infections.
